# Microbiome-Mitochondria Communication in the Regulation of Host Longevity

**DOI:** 10.1093/geroni/igaa057.2637

**Published:** 2020-12-16

**Authors:** Meng Wang

**Affiliations:** Baylor College of Medicine/HHMI, Houston, Texas, United States

## Abstract

Mitochondria are ancient relatives of bacteria in eukaryotic cells and dynamically interconnected through organelle fusion and fission. Given the close relationship between bacteria and eukaryotic mitochondria during evolution, my group is interested in understanding the critical role of their communication in regulating host’s longevity. We have conducted genome-scale screens to decipher how bacterial genetic composition impacts host longevity, leading to the discovery of specific bacteria-secreted metabolites that fine-tune the mitochondrial fusion-fission balance and consequently promotes longevity across different host species. We have further developed optogenetic approaches to manipulate bacterial gene expression and metabolite production inside the gut of live organisms, in order to investigate the microbiome-mitochondria communication in time and space. Our studies demonstrate a novel mode of signaling communication between bacteria and mitochondria, reveal its vital impacts on host healthy aging, and provide new methods to decipher the spatiotemporal relationship between the microbiome and the host.

